# The Chemical Constituents From Borojo and Their Anti‐Inflammatory Activity

**DOI:** 10.1002/cbdv.71712

**Published:** 2026-09-20

**Authors:** Jinying Ou, Zibin Lu, Lifang Zou, Yuanru Zheng, Xuan Zhao, Xiduan Wei, Linzhong Yu, Huihui Cao, Huhu Zeng, Chunlin Fan, Junshan Liu

**Affiliations:** ^1^ Third Level Research Laboratory of State Administration of Traditional Chinese Medicine, Guangdong Provincial Key Laboratory of Chinese Medicine Pharmaceutics, School of Traditional Chinese Medicine Southern Medical University Guangzhou China; ^2^ International Joint Laboratory of Zebrafish Models of Human Diseases and Drug Discovery, School of Traditional Chinese Medicine Southern Medical University Guangzhou China; ^3^ Guangdong Province Key Laboratory of Pharmacodynamic Constituents of TCM and New Drugs Research, College of Pharmacy Jinan University Guangzhou China; ^4^ Guangdong Basic Research Center of Excellence for Integrated Traditional and Western Medicine For Qingzhi Diseases Southern Medical University Guangzhou China

**Keywords:** anti‐inflammation, borojo, cyclopentenes, fused ring compound, zebrafish

## Abstract

Borojo, a fruit native to South America, remains to be comprehensively evaluated for its chemical profile. In this study, we isolated and purified nine compounds from borojo. Among these, three were newly identified cyclopentenes (**BLH‐1–BLH‐3**), one was a novel fused‐ring compound (**BLH‐4**), and the remaining five were known compounds (**BLH‐5–BLH‐9**). To assess their anti‐inflammatory potential, we evaluated the compounds in vitro using LPS‐stimulated RAW 264.7 murine macrophages. Additionally, we conducted in vivo experiments using zebrafish inflammation models induced by LPS, CuSO_4_, or tail transection. Our results showed that **BLH‐1**, **BLH‐5**, and **BLH‐9** significantly inhibited LPS‐induced TNF‐α and IL‐6 production (*p *≤ 0.05). Additionally, **BLH‐1**, **BLH‐2**, **BLH‐4**, **BLH‐6**, and **BLH‐7** effectively suppressed neutrophil recruitment in zebrafish triggered by LPS, CuSO_4_, or physical injury. Further mechanistic investigation in LPS‐induced RAW 264.7 cells revealed that **BLH‐1** reduced the expression levels of p‐IκBα and p‐STAT3 and inhibited nuclear translocation of NF‐κB p65. Taken together, these findings highlight borojo as a promising natural source of anti‐inflammatory agents and offer new directions for the discovery of bioactive, food‐derived anti‐inflammatory compounds.

## Graphical abstract

Three new cyclopentenes and one new fused‑ring compound were isolated from borojo. BLH‑1, BLH‑5, and BLH‑9 inhibited TNF‑α and IL‐6 release, while BLH‑1, BLH‑2, BLH‑4, BLH‑6, and BLH‑7 reduced neutrophil migration in zebrafish models. BLH‑1 was found to suppress NF‑κB and STAT3 pathways, suggesting borojo is a promising source of anti‑inflammatory compounds.

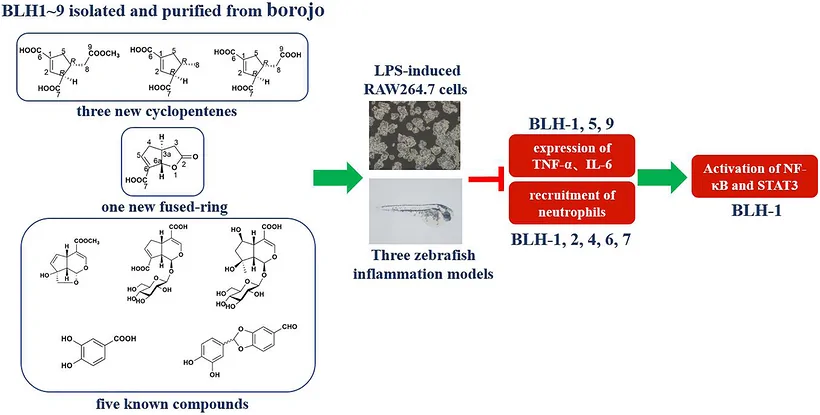

## 1 Introduction

Borojo (genus *Borojoa*, family Rubiaceae) is a tropical fruit native to the rainforests of Colombia, Brazil, Ecuador, and Panama. It includes the species *Borojoa patinoi* and *Borojoa sorbilis* [1]. Known for being energy‐dense and nutrient‐rich, it has long been used in traditional medicine for its purported antihypertensive, diuretic, and aphrodisiac effects [2]. Several classes of bioactive compounds, including organic acids (e.g., ascorbic and oxalic acids), triterpenes, flavonoids, and phenolic compounds, have been identified from borojo [3, 4]. However, cyclopentenes and their fused‑ring analogs have not been reported to date. Indeed, cyclopentenes have only been detected as synthetic intermediates, with no associated biological activity studies reported yet. Certain polyphenols present in the fruit have been linked to antimicrobial activity against pathogens including *Escherichia coli* and *Staphylococcus aureus* [4]. Given its notable nutritional and functional properties, borojo is under consideration for approval as a novel food in the European Union. Nevertheless, a systematic phytochemical investigation of borojo remains lacking. This limits a deeper understanding of its full pharmacological potential.

Despite its traditional use and nutritional relevance, the potential anti‑inflammatory properties of borojo remain largely unexplored. Inflammation is closely associated with many of the pathological conditions that borojo has been traditionally used to alleviate. Therefore, investigating its anti‑inflammatory activity is of considerable interest. Acute inflammation serves as a vital defense mechanism that helps restore tissue integrity after infection or injury. This tightly regulated process is essential for maintaining physiological balance. However, when it becomes excessive or fails to resolve, it can contribute to various pathological conditions such as endotoxemia, acute lung injury, and sepsis [5, 6]. Recent studies have identified new anti‑inflammatory compounds from medicinal plants. *Inula britannica* afforded a dimeric sesquiterpenoid that inhibited IKKβ/NF‑κB and activated Nrf2. *Euphorbia antiquorum* yielded an ingenane diterpenoid that similarly suppressed NO, COX‑2, and IL‑6 production. *Tripterygium wilfordii* provided a potent diterpenoid (IC_50_ 0.77 µM) that inhibited macrophage polarization [7, 8, 9]. It is evident that natural products offer a promising source for the discovery of anti‑inflammatory agents.

The zebrafish (*Danio rerio*) has emerged as a valuable vertebrate model for in vivo screening of anti‐inflammatory compounds. Its small size, rapid development, and optical transparency—especially in larvae—allow real‐time observation of immune cell dynamics during inflammation. Furthermore, zebrafish share a highly conserved innate immune system with mammals, including key inflammatory mediators, receptors, and effector cells [10]. Established models of inflammation in zebrafish, such as tail transection, CuSO_4_ exposure, or LPS exposure, offer robust platforms for high‐throughput compound testing [11, 12]. These systems have already proven effective in identifying anti‐inflammatory modulators with translational potential to mammalian models. This highlights the utility of zebrafish as a cost‑effective and physiologically relevant screening tool [13].

Given that the chemical constituents and pharmacological activities of borojo remain largely unexplored, we hypothesized that this fruit contains structurally diverse compounds with potential anti‑inflammatory properties. Therefore, this study aimed to isolate and characterize the chemical constituents from borojo, evaluate their anti‑inflammatory activity using in vitro and in vivo models, and preliminarily explore the mechanisms underlying the active compounds. From borojo, we isolated three novel cyclopentenes and one new fused‐ring compound. Their anti‐inflammatory activity was assessed in vitro by measuring LPS‐induced TNF‐α and IL‐6 production in macrophages, as well as in vivo using established zebrafish inflammation models.

## 2 Materials and Methods

### 2.1 General Experimental Procedure

IR spectra were acquired using a JASCO FT/IR‐480 Plus Fourier Transform infrared spectrometer (KBr pellet, JASCO Corporation, Japan). UV/Vis spectrum was obtained using a JASCO V‐550 ultraviolet‐visible spectroscopy (JASCO Corporation, Japan). Optical rotation was measured on a JASCO P‐1020 polarimeter (JASCO Corporation, Japan). ^1^H, ^13^C, and 2D NMR spectra were recorded using Bruker AV‐400 and Bruker AV‐500 NMR spectrometers (TMS is internal standard, Bruker, Germany). High‐resolution electrospray ionization mass spectrum (HRESIMS) analysis was carried out using an Agilent 6210 LC/MSD TOF mass spectrometer (Agilent, USA). High‐performance liquid chromatography (HPLC) separation was conducted on an Agilent 1260 Analytical HPLC system with COSMOSIL C_18_‐MS‐II column (4.6 × 250 mm, 5 µm) and COSMOSIL C_18_ column (20 × 250 mm, 5 µm). Column chromatography was performed using silica gel (100∼200 mesh and 200∼300 mesh, Qingdao Marine Chemical Plant, China), ODS (YMC Co., Ltd., Japan), and Sephadex LH‐20 gel (GE Healthcare, USA). Fractions were operated by silica gel GF_254_ pre‐coated plate for TLC (Yantai Chemical Industry Research Institute, China) or RP‐18 high silica gel pre‐coated plate for TLC (Merck, USA).

### 2.2 Chemicals and Materials

HPLC grade methanol and acetonitrile were obtained from Merck (USA). All other organic solvents used in the column chromatography were analytical grade (Tianjin Damao Chemical Plant, Tianjin, China). The freeze‐dried powder of *B. sorbilis* Cuatrec fruit was supplied by South America Ecuador Oriental Food Company in July 2015. The source plant of the medicinal material was taxonomically authenticated as *B. sorbilis* Cuatrec by Professor Guangxiong Zhou from the College of Pharmacy at Jinan University. The corresponding voucher specimen (No. 20150801) is deposited in the Institute of Traditional Chinese Medicine and Natural Products of Jinan University. Fetal bovine serum (FBS) and Dulbecco's modified Eagle medium (DMEM) were purchased from Thermo Fisher Scientific (Waltham, USA). The enzyme‐linked immunosorbent assay (ELISA) kit for TNF‐α and IL‐6 was obtained from Dakewei (Beijing, China). Dexamethasone (Dex) was obtained from Tianxin (Guangzhou, China). Tricaine was obtained from Macklin (Shanghai, China), and CuSO_4_ was purchased from Guangzhou Chemical Reagent Factory (Guangzhou, China). LPS (*E. coli*, O55: B5, L2880) and other reagents were obtained from Sigma–Aldrich (St. Louis, USA).

### 2.3 Isolation and Purification

A freeze‐dried powder of *B. sorbilis* Cuatrec fruit weighing 6500 g was suspended in water and successively extracted with ethyl acetate and *n*‐butanol. The resulting extraction solution was concentrated under vacuum to obtain the ethyl acetate extract (36 g), *n*‐butanol extract (550 g), and water extract. The ethyl acetate extract (36 g) was subjected to silica gel column chromatography (CC) (gradient system of petroleum ether/ethyl acetate, 100:0 to 0:100), resulting in the isolation of ten main fractions (Fr.1∼Fr.10). Fr.4 (2.5 g) was further subjected to ODS CC with MeOH‐H_2_O (90:10 to 100:0), yielding seven subfractions (Fr.4a∼Fr.4 g). Subsequently, Fr.4c, Fr.4d, and Fr.4e were subjected to Sephadex LH‐20 gel CC, preparative HPLC, and recrystallization to obtain **BLH‐2** (10 mg) and **BLH‐8** (20 mg). Fr.6 (2.6 g) was subjected to ODS CC with MeOH‐H_2_O (95:5 to 100:0) to obtain six subfractions (Fr.6a ∼ Fr.6f). Fr.6c and Fr.6d were subjected to Sephadex LH‐20 gel CC and preparative HPLC to obtain **BLH‐4** (20 mg) and **BLH‐9** (12 mg). Fr.7 (2.5 g) was eluted with ODS CC with MeOH‐H_2_O (95:5 to 100:0) to yield ten subfractions (Fr.7a∼ Fr.7j). Finally, Fr.7d, Fr.7e, and Fr.7f were subjected to Sephadex LH‐20 gel CC and preparative HPLC to obtain **BLH‐1** (10 mg), **BLH‐3** (12 mg), **BLH‐5** (17 mg), **BLH‐6** (200 mg), and **BLH‐7** (9 mg).

#### 2.3.1 BLH‐1

Yellow oil; ${\left[\alpha \right]}_{D}^{25}$ = + 8.1 (*c =* 0.93, MeOH); UV (MeOH) *λ*_max_: 213 nm; IR *v*_max_ (KBr): 3450, 1718, 1637 cm^−1^; HR–ESI–MS *m/z* 251.0520 [M+Na]^+^ (C_10_H_12_O_4_Na, calc. 251.0526); ^1^H NMR (CD_3_OD, 400 MHz) and ^13^C NMR (CD_3_OD, 100 MHz) data see Table 1.

**TABLE 1:** NMR data of **BLH‐1** (MeOH, *δ*, *J* in Hz).

No.	^1^H	^13^C	^1^H‐^1^H COSY	HMBC
1		136.1		
2	6.86 (br s)	146.4	H‐3, 5	C‐1, 3, 4, 5, 6,
3	3.48 (d, 5.9)	56.5	H‐2, 4	C‐1, 2, 4, 5, 7, 8
4	2.86 (m)	41.4	H‐3, 5, 8	C‐1, 2, 3, 5, 7, 8, 9
5	2.88 (m); 2.31 (m)	39.7	H‐2, 4	C‐1, 2, 3, 4, 6, 7, 8; C‐1, 2, 4, 6, 7, 8
6		167.2		
7		176.3		
8	2.54 (qd, 16.1, 6.9)	40.3	H‐4	C‐3, 4, 5, 9
9		175.5		
10	3.68 (s)	52.6		C‐9

#### 2.3.2 BLH‐2

Brownish yellow amorphous powder; ${\left[\alpha \right]}_{D}^{25}$ = + 8.5 (*c* = 0.91, MeOH); UV (MeOH) *λ*_max_: 214 nm; IR *v*_max_ (KBr): 2965, 1707, 1682 cm^−1^; HR–ESI–MS *m/z* 193.0479 [M+Na]^+^ (C_8_H_10_O_4_Na, calc. 193.0471); ^1^H NMR (CD_3_OD, 500 MHz) and ^13^C NMR (CD_3_OD, 125 MHz) data see Table 2.

**TABLE 2:** NMR data of **BLH‐2** (MeOH, *δ*, *J* in Hz).

No.	^1^H	^13^C	^1^H‐^1^H COSY	HMBC
1		136.4		
2	6.83 (dd, 4.5, 2.4)	146.4	H‐3, 5	C‐1, 3, 4, 5, 6
3	3.26 (qd, 5.7, 2.0)	58.8	H‐2, 4	C‐1, 2, 4, 5, 7, 8
4	2.57 (m)	40.1	H‐3, 5, 8	C‐1, 2, 3, 5, 7, 8
5	2.81 (ddt, 18.5, 8.0, 2.5); 2.16 (ddt, 18.5, 5.5, 2.5)	41.7	H‐2, 4	C‐1, 2, 3, 4, 8; C‐1, 2, 4, 8
6		167.9		
7		178.2		
8	1.19 (d, 6.5)	21.4	H‐4	C‐3, 4, 5

#### 2.3.3 BLH‐3

Yellow oil; ${\left[\alpha \right]}_{D}^{25}$ = + 8.9 (*c =* 0.39, MeOH); UV (MeOH) *λ*_max_: 215 nm; IR *v*_max_ (KBr): 3078, 1695, 1633 cm^−1^; HR–ESI–MS *m/z* 237.0377 [M+Na]^+^ (C_9_H_10_O_6_Na, calc. 237.0370); ^1^H NMR (CD_3_OD, 500 MHz)and ^13^C NMR (CD_3_OD, 125 MHz) data see Table 3.

**TABLE 3:** NMR data of **BLH‐3** (MeOH, *δ*, *J* in Hz).

No.	^1^H	^13^C	^1^H‐^1^H COSY	HMBC
1		136.1		
2	6.85 (br s)	146.2	H‐3, 5	C‐1, 3, 4, 5, 6, 7
3	3.44 (m)	56.5	H‐2, 4, H_b_‐5	C‐1, 2, 4, 5, 7, 8
4	2.86 (m)	41.2	H‐3, 5, 8	C‐1, 2, 3, 5, 7, 8, 9
5	2.88 (m) 2.31 (m)	39.7	H‐2, 4	C‐1, 2, 3, 4, 6, 7, 8; C‐1, 2, 4, 6, 7, 8
6		167.5		
7		177.6		
8	2.55 (m)	40.3	H‐4	C‐3, 4, 5, 9
9		175.6		

#### 2.3.4 BLH‐4

Yellow oil; ${\left[\alpha \right]}_{D}^{25}$ = + 5.4 (*c =* 0.98, MeOH); UV (MeOH) *λ*_max_: 211 nm; IR *v*_max_ (KBr): 3466, 1762, 1635 cm^−1^; HR–ESI–MS *m/z* 191.0318 [M+Na]^+^ (C_8_H_8_O_4_Na, calc. 191.0315); ^1^H NMR (CD_3_OD, 500 MHz)and ^13^C NMR (CD_3_OD, 125 MHz) data see Table 4.

**TABLE 4:** NMR data of **BLH‐4** (MeOH, *δ*, *J* in Hz).

No.	^1^H	^13^C	^1^H‐^1^H COSY	HMBC
2		179.6		
3	2.96 (m), 2.43 (m)	36.3	H‐3a	C‐2, 3a, 4, 6a
3a	3.31 (m)	37.1	H‐3, 4, 6a	C‐2, 3, 4, 5, 6
4	2.97 (m), 2.49 (m)	40.5	H‐3a, 5	C‐2, 3, 3a, 6a, 7
5	7.10 (t, 2.4)	151.0	H‐4	C‐3, 3a, 4, 6a, 7
6		136.3		
6a	5.69 (dd, 7.5, 2.0)	89.2	H‐3a, H_b_‐4	C‐2, 3, 3a, 4, 5, 6
7		166.6		

### 2.4 Cell Culture

Murine macrophage RAW 264.7 cells (RRID: CVCL_0493) were obtained from the American Type Culture Collection (ATCC, Manassas, VA, USA). The cells were cultured in DMEM (high glucose, Thermo–Fisher Scientific, Waltham, USA) supplemented with 10% (*v*/*v*) FBS (Thermo–Fisher Scientific, Waltham, USA) and 1% (*v*/*v*) penicillin/streptomycin (100 U/mL penicillin and 100 µg/mL streptomycin, Gibco). The culture was maintained in a 5% CO_2_ incubator at 37°C. Cultures were passaged every 2–3 days upon reaching 80%–90% confluence using 0.25% trypsin‐EDTA (Gibco), and cells from passages 5 to 20 were used for all experiments.

### 2.5 Cell Viability Assay

Cell viability was assessed using the MTT assay. RAW 264.7 cells were seeded at a density of 1 × 10^4^ cells per well in a 96‐well plate. After 24 h of incubation, **BLH‐1**–**BLH‐9** at different concentrations (10, 20 µM) were added to each well, followed by an additional 24 h incubation. Subsequently, 20 µL of MTT dye (5 mg/mL) was added and incubated for another 4 h. The incubation buffer was discarded, and 100 µL of dimethyl sulfoxide was used to dissolve the formazan crystals [14]. The absorbance at 490 nm was measured using a microplate reader (Thermo–Fisher Scientific).

### 2.6 ELISA

RAW 264.7 cells were seeded in a 96‐well plate at a density of 1 × 10^4^ cells per well. After 24 h of incubation, the cells were pre‐treated with **BLH‐1**–**BLH‐9** (10, 20 µM) for 24 h. Subsequently, the cells were exposed to LPS (100 ng/mL) for 9 h. Following incubation, the culture medium was collected to determine TNF‐α and IL‐6 production using an ELISA kit as described previously [15].

### 2.7 LPS‐Induced Zebrafish Inflammation Model

All experiments were conducted in accordance with the ARRIVE guidelines for the care and use of laboratory animals, approved by the Ethical Committee of the Southern Medical University (no. L2022087). Transgenic zebrafish Tg (*mpo*:GFP) expressing green fluorescent protein in neutrophils were cultivated in the Key Laboratory of Zebrafish Modeling and Drug Screening for Human Diseases Institute at School of Traditional Chinese Medicine, Southern Medical University (Guangzhou, China). A constant temperature of 28.5°C and a 14 h light/10 h dark cycle were used for zebrafish maintenance. The fish were given dry larval diets and brine shrimp thrice daily. For embryo collection, natural spawning in breeding tanks was employed, and the resulting embryos were raised at 28.5°C in egg water that included 0.02 mg/mL methylene blue (Meilunbio, Dalian, China).

The experimental unit is defined as each individual embryo/larva. All experiments were performed with three independent biological replicates. 3 days post‐fertilization (dpf) zebrafish larvae were anesthetized using 0.02% tricaine. To induce an inflammation model, 2 nL LPS (2 mg/mL) was microinjected into the yolk [16]. Phosphate buffered saline (PBS) was used as a negative control. To evaluate the anti‐inflammatory activity of **BLH‐1**–**BLH‐9** in the LPS‐induced zebrafish inflammation model, larvae were treated with **BLH‐1**–**BLH‐9** (20 µM) or Dex (5 µg/mL) dissolved in egg water by immersion. The treatment solutions were freshly prepared and renewed every 6 h to maintain stable concentrations. After 12 h, the larvae were collected for neutrophil observation.

All observations were performed blind to treatment groups.

### 2.8 CuSO_4_‐Induced Zebrafish Inflammation Model

The same rearing and handling conditions as described in Section 2.7 were applied. To induce acute inflammation, 3 dpf larvae were co‑incubated in egg water containing CuSO_4_ (20 µM) together with either **BLH‑1–BLH‑9** (20 µM) or Dex (2 µg/mL). A vehicle control (egg water with an equivalent volume of solvent) was included in parallel. Because the incubation lasted only 2 h, the solution was prepared fresh and not renewed during this short period. After 2 h of cultivation, the larvae were anaesthetised and observed, and the neutrophils in lateral line neuromast areas were counted under an MVX10 fluorescence microscope (Olympus, Tokyo, Japan). All counting was performed in a blinded manner.

### 2.9 Tail Transection‐Induced Zebrafish Inflammation Model

The 3 dpf zebrafish were anesthetized, and their tails were transected using a stereomicroscope (Olympus, SZX7, Tokyo, Japan) to induce injury. The experiments were repeated three independent times. Immediately after transection, the zebrafish were treated with **BLH‐1**–**BLH‐9** (20 µM) or Dex (2 µg/mL) for 6 h. A vehicle control (egg water plus solvent) was processed identically. The neutrophils in the injured areas were observed using an MVX10 fluorescence microscope (Olympus, Tokyo, Japan). The number of neutrophils at the wound margin was quantified using ImageJ software. Quantification was performed by an observer blinded to the experimental groups.

### 2.10 Western Blotting and Confocal Microscopy

RAW 264.7 cells were seeded in a 60 mm dish at a density of 6 × 10^5^ or in a glass dish at a density of 2 × 10^5^ and cultured for 24 h. The cells were then pretreated with **BLH‑1** (50 µM) for 24 h, followed by co‑incubation with LPS (100 ng/mL) for an additional 9 h. For Western blotting, the protein levels of IκBα and p‐IκBα (Ser32) were measured using Western blotting using antibodies against IκBα and p‑IκBα (Ser32) at a 1:1000 dilution, with β‑actin serving as the internal reference, and the nuclear translocation of NF‐κB p65 and the expression of p‐STAT3 (Tyr705) were examined using confocal microscopy. Western blotting and confocal microscopy procedures were carried out following previously described methods [17].

### 2.11 Statistical Analysis

All data are presented as mean ± standard deviation (SD). For in vitro cellular assays (Table 5 and Figure 3), each experiment was independently repeated at least three times (*n *= 3). For in vivo zebrafish inflammation models (Figure 2), each group consisted of 8 larvae (*n *= 8). For comparisons between two groups, a two‐tailed Student's *t*‐test was applied. For comparisons among three or more groups, a one‐way analysis of variance (ANOVA) was performed, followed by Tukey's honestly significant difference *post‐hoc* test. All statistical analyses were conducted using SPSS 20.0 software (IBM, Armonk, NY, USA). A probability value of *p *≤ 0.05 was considered statistically significant.

**TABLE 5:** Anti‐inflammatory effects on LPS‐stimulated RAW 264.7 cells (*n *= 3).

No.	Concentration	TNF‐α (ng/ml)	IL‐6 (ng/ml)
LPS	100 ng/ml	85.233 ± 3.552	7.839 ± 0.445
**BLH‐1**	20 µM	42.443 ± 15.325^***^	3.849 ± 0.225^**^
	10 µM	88.664 ± 14.514	6.984 ± 1.342
**BLH‐2**	20 µM	90.714 ± 1.971	7.989 ± 0.541
	10 µM	88.663 ± 8.976	8.093 ± 0.548
**BLH‐3**	20 µM	105.857 ± 4.112	8.325 ± 0.452
	10 µM	87.615 ± 11.225	7.964 ± 0.673
**BLH‐4**	20 µM	87.373 ± 1.425	7.367 ± 0.482
	10 µM	98.568 ± 1.982	7.954 ± 0.346
**BLH‐5**	20 µM	47.390 ± 6.806^***^	2.845 ± 0.343^**^
	10 µM	47.410 ± 1.947^***^	4.895 ± 0.497^**^
**BLH‐6**	20 µM	90.560 ± 4.254	6.985 ± 0.543
	10 µM	76.619 ± 4.376	7.488 ± 0.408
**BLH‐7**	20 µM	63.602 ± 4.791	6.745 ± 0.475
	10 µM	68.835 ± 2.649	6.989 ± 0.378
**BLH‐8**	20 µM	69.100 ± 1.140	7.046 ± 1.021
	10 µM	68.569 ± 14.164	7.954 ± 0.938
**BLH‐9**	20 µM	60.793 ± 0.367^*^	5.961 ± 0.243^**^
	10 µM	78.878 ± 4.074	7.566 ± 0.573

## 3 Results and Discussion

### 3.1 Structural Elucidation of the Isolated Compounds

The structural assignments of **BLH‐1**–**BLH‐4** were based on the 1D and 2D NMR data shown in Figures S1–S22, with the epimer structures illustrated therein. **BLH‐1**, ${\left[\alpha \right]}_{D}^{25}$ = + 8.1 (*c* = 0.93, MeOH), was obtained as a yellowish oil. The molecular formula was established as C_10_H_12_O_4_ by HRESIMS at *m*/*z* of 251.0520 [M + Na]^+^ (calcd for C_10_H_12_O_4_Na, 251.0526). IR spectra indicated the presence of hydroxyl (3450 cm^−1^), carbonyl (1718 cm^−1^), and double bond (1637 cm^−1^) moieties. The ^1^H NMR spectrum (Figure S1) displayed one olefinic proton [*δ*_H_ 6.86 (1H, br s, H‐2)], two methines [*δ*_H_ 3.48 (1H, d, *J *= 5.9 Hz, H‐3) and 2.86 (1H, m, H‐4)], two methylenes [*δ*_H_ 2.88 (1H, m, H‐5a), 2.31 (1H, m, H‐5b), and 2.54 (2H, qd, *J *= 16.1, 6.9 Hz, H_2_‐8)], and one methoxyl [*δ*_H_ 3.68 (3H, s, H_3_‐10)]. The ^13^C NMR and DEPT‐135 spectrum (Figure S2) revealed 10 carbon signals, including three carbonyls [*δ*_C_ 176.3 (C‐7), 175.5 (C‐9) and 167.2 (C‐6)], a pair of olefinic bonds [*δ*_C_ 146.4 (C‐2) and 136.1 (C‐1)], and one methoxyl [*δ*_C_ 52.6 (C‐10)].

**FIGURE 1:**
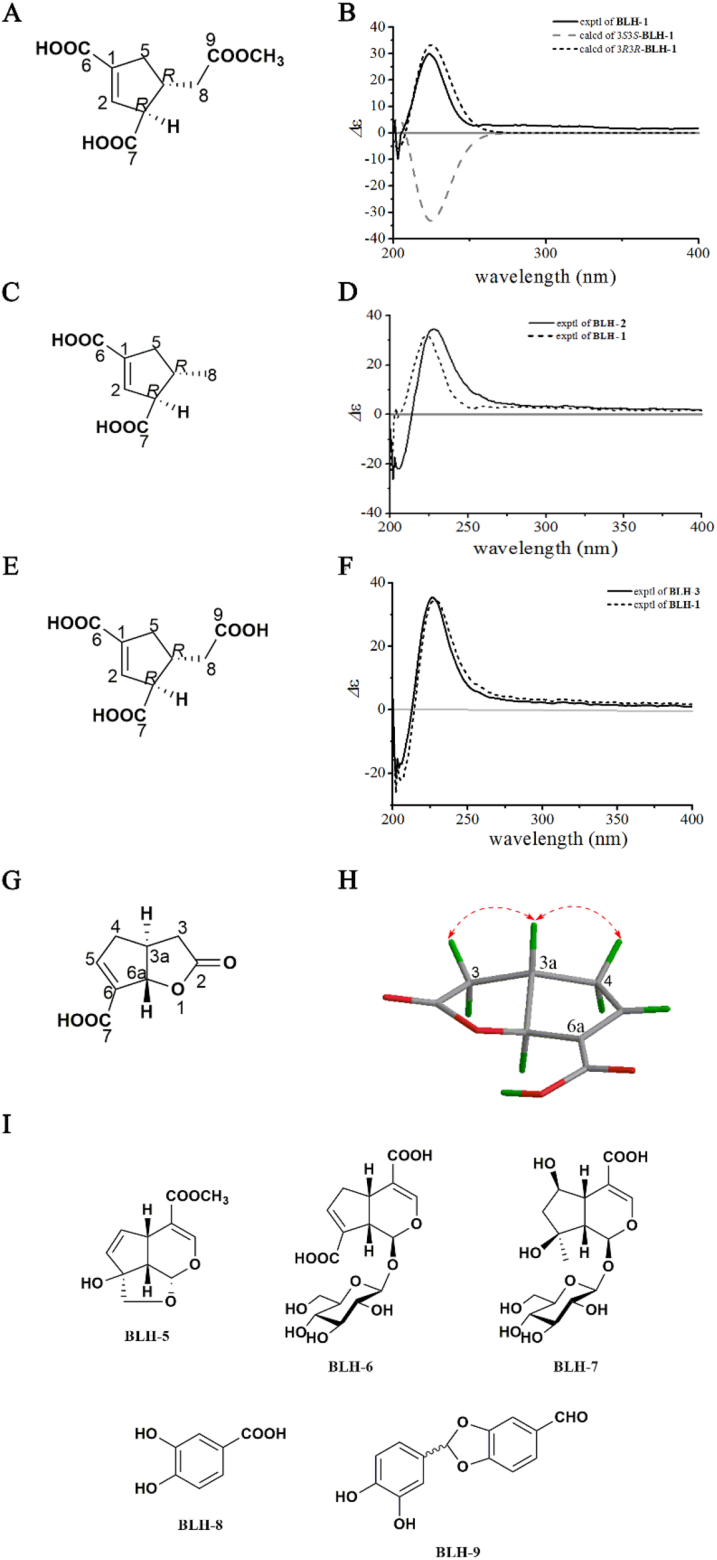
Structure (A) and calculated and experimental CD spectra (B) of **BLH‐1**. Structure of **BLH‐2** (C) and experimental CD spectra of **BLH‐2** and **BLH‐1** (D). Structure of **BLH‐3** (E) and experimental CD spectra of **BLH‐3** and **BLH‐2** (F). Structure of **BLH‐4** (G) and key NOESY correlations of **BLH‐4** (H). Structures of **BLH‐5**, **6**, **7**, **8**, **9** (I).

The key ^1^H‐^1^H COSY correlations of H_2_‐5/H‐4/H‐3/H‐2 together with the HMBC cross‐peaks from H‐2 to C‐5, and from H‐3/H‐4 to C‐1 established a cyclopentene structural unit. Additionally, the ^1^H‐^1^H COSY connectivity between H‐4 and H‐8, along with the HMBC correlations from H‐8/H‐10 to C‐9 and from H‐3/H_2_‐5 to C‐8, defined a methoxycarbonylmethyl moiety attached to C‐4. Furthermore, the HMBC cross‐peaks from H‐2 to C‐6 and from H‐4 to C‐7 suggested the presence of two carboxylic acid groups at C‐1 and C‐3, respectively. Thus, the planar structure of **BLH‐1** was established as shown in Figure 1A.

The relative configuration of **BLH‐1** was assigned as (±)‐*trans*‐4‐(2‐methoxy‐2‐oxoethyl) cyclopent‐2‐ene‐1,3‐dicarboxylic acid by comparison with structurally related cyclopentene dicarboxylic acids, namely (±)‐*trans*‐4‐methylcyclopent‐3‐ene‐1,2‐dicarboxylic acid and (±)‐*cis*‐4‐methylcyclopent‐3‐ene‐1,2‐dicarboxylic acid (Figure S9). These reference compounds were previously synthesized and characterized by Hamersak et al. [18]. In these reference compounds, the relative stereochemistry at C‐3 and C‐4 is reliably reflected by the vicinal coupling constant between H‐3 and H‐4, with reported ^3^ *J*_H3–H4_ values of approximately 7.0 Hz for the trans isomer and 9.0 Hz for the cis isomer. In the ^1^H NMR spectrum of **BLH‐1**, a ^3^ *J*_H3–H4_ value of 5.9 Hz was observed, supporting a trans relationship between C‐3 and C‐4.

The absolute configuration of **BLH‐1** was assigned by comparing the experimental CD spectrum with the calculated spectra for the (3*S*,4*S*)‐ and (3*R*,4*R*)‐enantiomers (Figure 1B). The experimental CD spectrum showed good agreement with the calculated curve of (3*R*,4*R*)‐**BLH‐1**. Therefore, **BLH‐1** was characterized as a new cyclopentene derivative (3*R*,4*R*)‐4‐(2‐methoxy‐2‐oxoethyl)cyclopent‐2‐ene‐1,3‐dicarboxylic acid, named borojenedioic acid A.

**BLH‐2**, ${\left[\alpha \right]}_{D}^{25}$ = + 8.5 (*c* = 0.91, MeOH) was isolated as a brownish‐yellow amorphous powder. Its molecular formula was assigned as C_8_H_10_O_4_ based on the HRESIMS at *m*/*z* 193.0479 [M + Na]^+^ (calcd for C_8_H_10_O_4_Na 193.0471). The IR spectrum exhibited characteristic absorption bands for hydroxyl (2965 cm^−1^), carbonyl (1708 cm^−1^), and double bond (1682 cm^−1^) groups. Detailed analysis of the 1D and 2D NMR data suggested that the structure of **BLH‐2** was closely related to that of **BLH‐1**, with the primary difference being the presence of a methyl group attached to C‐4 in **BLH‐2** instead of the methoxycarbonylmethyl moiety in **BLH‐1**. This substitution was further supported by the ^1^H‐^1^H COSY correlations of H‐8 (*δ*_H_ 1.19, d, *J *= 6.5 Hz)/H‐4 (*δ*_H_ 2.57, m)/H‐3 (*δ*_H_ 3.26, qd, *J *= 5.7, 2.0 Hz)/H‐2 (*δ*_H_ 6.83, dd, *J *= 4.5, 2.4 Hz) and the HMBC cross‐peaks from H‐8 to C‐3 (*δ*_C_ 58.8)/C‐5 (*δ*_C_ 41.7). Likewise, the absolute configuration of **BLH‐2** was determined to be (3*R*,4*R*) by comparing its experimental spectrum with the calculated CD curve. Thus, **BLH‐2** was assigned as a new cyclopentene derivative (3*R*,4*R*)‐4‐methylcyclopent‐2‐ene‐1,3‐dicarboxylic acid, named borojenedioic acid B.

**BLH‐3**, ${\left[\alpha \right]}_{D}^{25}$ = + 8.1 (*c* = 0.93, MeOH) was afforded as a yellowish oil. Its molecular formula was established as C_9_H_10_O_6_ by HRESIMS at *m*/*z* 237.0377 [M + Na]^+^ (calcd for C_9_H_10_O_6_Na, 237.0370). Detailed interpretation of the 1D and 2D NMR data suggested that the structure of **BLH‐3** was highly similar to that of **BLH‐2**, differing primarily by the presence of an additional carboxyl group at C‐8. This structural change was confirmed by the key HMBC correlation from H‐4 (*δ*_H_ 2.86, m) to C‐9 (*δ*_C_ 175.6). Furthermore, the CD spectrum of **BLH‐3** closely resembled that of **BLH‐2** (Figure 1F), indicating that they share the same absolute configuration. Thus, **BLH‐3** was assigned as a new cyclopentene derivative (3*R*,4*R*)‐4‐(carboxymethyl) cyclopenten‐1‐ene,3‐dicarboxylic acid, named borojenedioic acid C.

**BLH‐4**, ${\left[\alpha \right]}_{D}^{25}$ = + 5.4 (*c* = 0.98, MeOH), was obtained as a yellowish oil. The HRESIMS gave the quasi‐molecular ion peak at *m*/*z* 191.0318 [M + Na]^+^ (calcd for C_8_H_8_O_4_Na 191.0315), which was consistent with the molecular formula C_8_H_8_O_4_. The IR spectrum showed absorption bands for hydroxyl (3466 cm^−1^), carbonyl (1762 cm^−1^), and double bond (1635 cm^−1^) groups. The ^1^H NMR spectrum (Figure S14) exhibited signals corresponding to one olefinic proton [*δ*_H_ 7.10 (1H, t, *J *= 2.4 Hz, H‐5)], one oxygenated methine [*δ*_H_ 5.69 (1H, dd, *J *= 7.5, 2.0 Hz, H‐6a)], one methane [*δ*_H_ 3.31 (1H, m, H‐3a)], and two methylenes [*δ*_H_ 2.97 and 2.49 (2H, m, H_2_‐4); 2.96 and 2.43 (2H, m, H_2_‐3)]. The ^13^C NMR data revealed 8 carbon resonances, which were attributed by DEPT‐135 and HSQC spectra to two carbonyls [*δ*_C_ 179.6 (C‐2) and 166.6 (C‐7)], a pair of olefinic bonds [*δ*_C_ 151.0 (C‐8) and 136.3 (C‐9)], one oxygenated methane [*δ*_C_ 89.2 (C‐6a)], one methane [*δ*_C_ 37.1 (C‐3a)] and two methylenes [*δ*_C_ 40.5 (C‐4) and 36.3 (C‐3)] (Table 4).

The ^1^H‐^1^H COSY connectivities for H‐5/H_2_‐4/H‐3a/H_2_‐3 and of H‐3a/H‐6a, combined with the HMBC cross‐peaks of H‐3a to C‐5/C‐2/C‐6 and H‐6a to C‐4/C‐3, enabled the construction of a cyclopenta[b]furan‐2‐one fused skeleton of **BLH‐4**. Further comparison indicated that **BLH‐4** closely resembled Corey lactone diol [19], except that **BLH‐4** contained a carboxylic acid group and a double bond in place of a hydroxyl group and a hydroxymethyl group. This assignment was supported by the chemical shifts of C‐5 and C‐6, and the HMBC cross‐peak of H‐5 to C‐7. NOESY correlations (Figure S18) between H‐3a and Ha‐3/Ha‐4 suggested these protons were co‐facial. The W‐type coupling between H‐6a and Hb‐4 indicated the two protons were on one side of the ring, whereas they are trans to H‐3a, Ha‐4, and Ha‐3 (Figure 1H). Collectively, **BLH‐4** was assigned as a new lactone *trans*‐2‐oxo‐3,3a,4,6a‐tetrahydro‐2*H*‐cyclopenta[b]furan‐5‐ carboxylic acid, named Borojofuranoic acid A.

To date, there have been no reports on the isolation of cyclopentenes and fused‐ring cyclopentenes from *Borojoa* or related species of the Rubiaceae family. These compounds have also not been identified in plants of any other families or genera; all available literature references describe their preparation via chemical synthesis only. Compared with the synthetic compounds reported in literature, BLH‐1 possesses a novel structure featuring a CH_2_COOCH_3_ group at the R_3_ position; BLH‐2 is structurally unique with a CH_3_ substituent at R_3_; and BLH‐3 exhibits an unprecedented skeleton bearing a CH_2_COOH moiety at R_3_. BLH‐4 features a novel structure with a COOH group at the R_1_ position, whereas the synthetic compounds documented in the literature bear a CH_2_OH substituent at R_1_ (Figures S23 and S24; Tables S1 and S2).

### 3.2 Anti‐Inflammatory Activities

TNF‐α and IL‐6 are key pro‐inflammatory cytokine that plays a central role in the initiation and amplification of inflammatory responses [20]. To exclude the possibility that the observed effects of the BLH compounds were attributable to cytotoxicity, the viability of RAW 264.7 macrophages treated with **BLH‐1**–**BLH‐9** was first evaluated using an MTT assay (structures of **BLH‐5**–**9** are shown in Figure 1I). As summarized in Table S3, the cell viability of all tested compounds was greater than 85% at concentrations of 10 or 20 µM. These concentrations were therefore selected for subsequent functional assays. The effects of **BLH‐1**–**BLH‐9** on inflammatory cytokine production were next examined in LPS‐stimulated RAW 264.7 cells by measuring TNF‐α and IL‐6 secretion. As expected, LPS exposure markedly increased TNF‐α and IL‐6 release. This increase was significantly attenuated by treatment with **BLH‐1** (20 µM), **BLH‐5** (10 and 20 µM), and **BLH‐9** (20 µM) (Table 5). These results indicate that these compounds possess inhibitory activity against LPS‐induced inflammatory signaling in macrophages.

**FIGURE 2:**
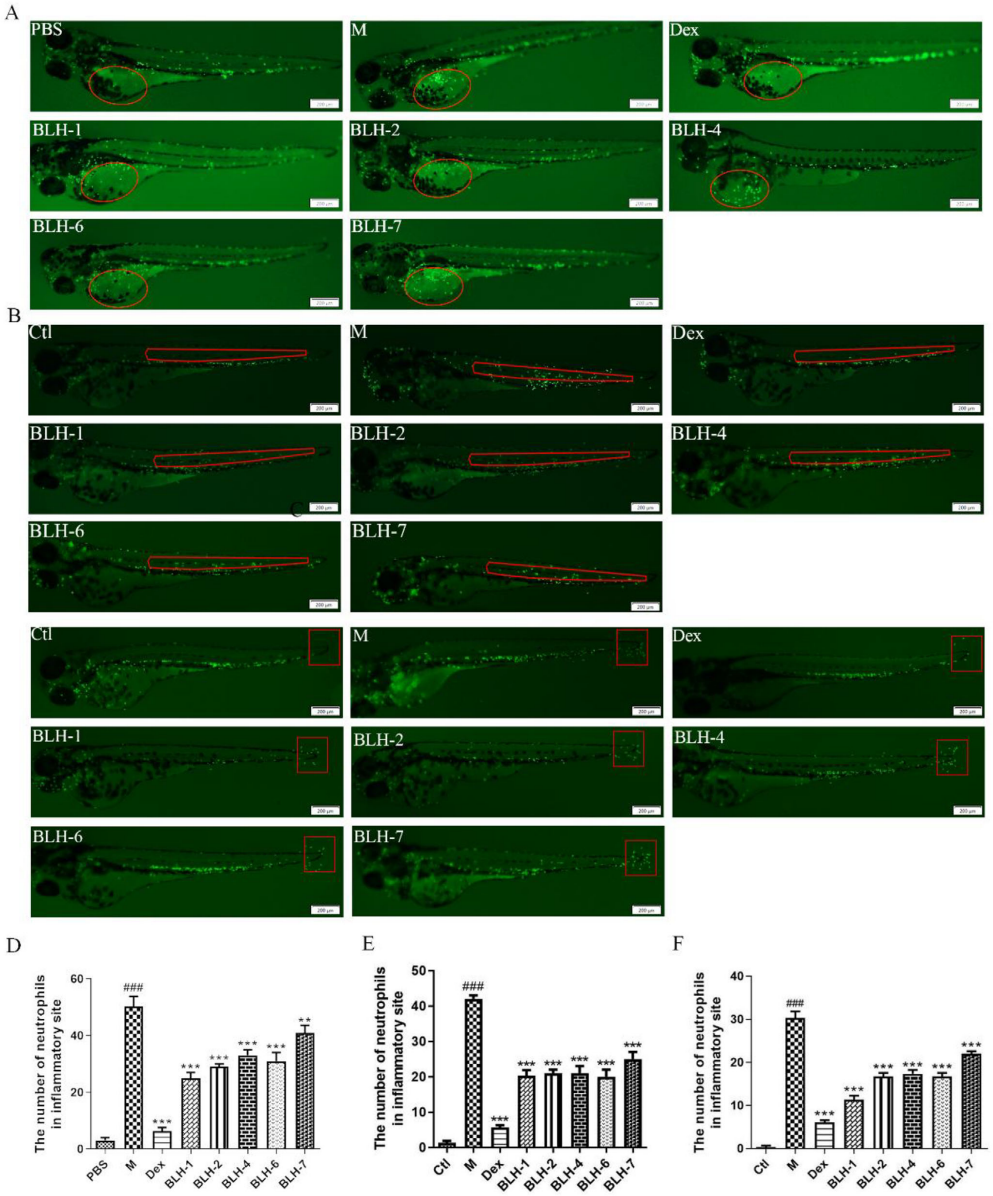
**BLH‐1, ‐2, ‐4, ‐6, ‐7** reduce neutrophil recruitment in zebrafish inflammation models induced by LPS, CuSO_4_, and tail transection zebrafish. (A) Fluorescence imaging of the yolk sac region (red frame) in LPS‐induced *Tg* (*mpo*:GFP) zebrafish. The scale bar represents 200 µm. (B) Fluorescence imaging of zebrafish in the CuSO_4_‐induced zebrafish inflammation model. The scale bar represents 200 µm. (C) Fluorescence imaging of zebrafish in the tail transection‐induced zebrafish inflammation model. The scale bar represents 200 µm. (D–F) Quantification of neutrophils in the region of interest in zebrafish inflammation models induced by LPS, CuSO_4_, and tail transection zebrafish. “PBS” referred to the control group, while “M” referred to the model group. Data were expressed as the mean ± SD, *n *= 8. Normality of the data distribution was assessed using the Shapiro–Wilk test, and homogeneity of variances was evaluated by Levene's test prior to statistical analysis. ^###^ *p* < 0.001 versus control group, ^**^ *p* < 0.01, ^***^ *p* < 0.001 versus model group by One‐way ANOVA with Tukey's test.

Neutrophils are critical regulators of inflammation and contribute to both the initiation and resolution of inflammatory responses. However, under pathological conditions, excessive neutrophil recruitment and activation can lead to sustained release of pro‑inflammatory mediators, thereby aggravating tissue injury. This is observed in LPS‑induced sepsis and severe viral infections such as COVID‑19 [21, 22, 23]. To evaluate the in vivo anti‐inflammatory effects of the BLH compounds, *Tg*(*mpo*:GFP) transgenic zebrafish larvae, in which neutrophils are fluorescently labeled, were employed. Due to limited compound availability, **BLH‐1, ‐2, ‐4, ‐6**, and **‐7** were selected for zebrafish experiments. Preliminary toxicity tests confirmed that these compounds did not cause mortality or evident inflammatory effects in zebrafish, and a concentration of 20 µM was therefore selected for subsequent experiments. An acute inflammation model was established by microinjecting LPS (2 mg/mL) into the yolk sac. LPS challenge induced pronounced neutrophil migration and accumulation at the injection site, whereas treatment with dexamethasone or **BLH‐1, ‐2, ‐4, ‐6**, and **‐7** markedly reduced neutrophil recruitment (Figure 2A,D). The anti‐inflammatory activities of these compounds were further validated using chemically and physically induced zebrafish inflammation models. Exposure to CuSO_4_, which causes damage to lateral line neuromast hair cells, and tail transection, which induces mechanical injury, both triggered robust neutrophil accumulation at the sites of injury (Figure 2B,C,E,F) [24, 25, 26]. In both models, treatment with **BLH‐1, ‐2, ‐4, ‐6**, and **‐7** significantly attenuated neutrophil migration, further supporting their in vivo anti‐inflammatory potential.

**FIGURE 3:**
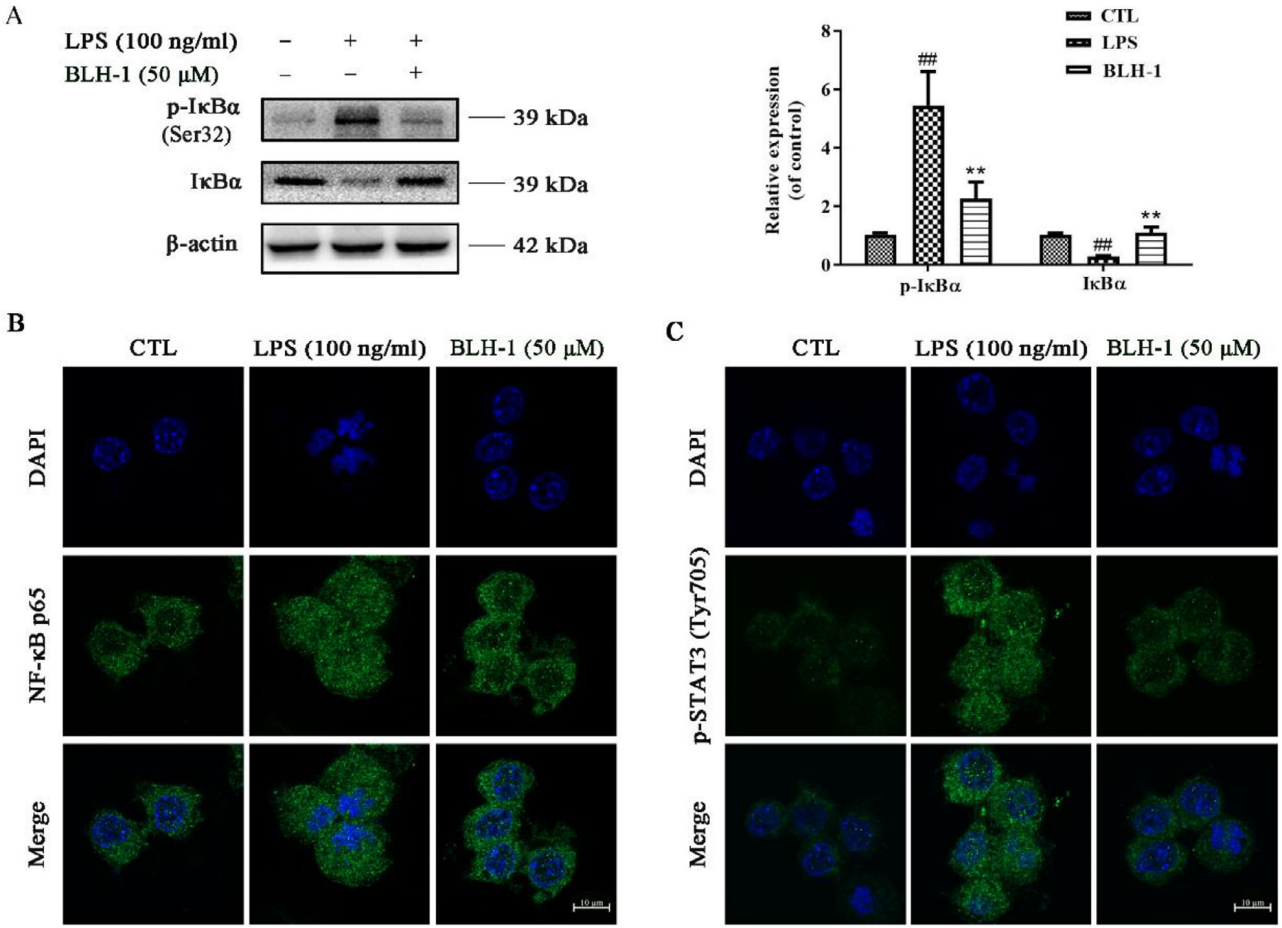
**BLH‐1** inhibits the NF‐κB and STAT3 signaling in RAW 264.7 cells. Cells were pretreated with BLH‐1 (50 µM) for 24 h, followed by co‐incubation with LPS (100 ng/mL) for another 9 h. (A) Protein expressions levels of IκBα and p‐IκBα (Ser32) assessed using Western blotting. Data were expressed as the mean ± SD, *n *= 3. Normality of the data distribution was assessed using the Shapiro–Wilk test, and homogeneity of variances was evaluated by Levene's test prior to statistical analysis. ^##^ *p* < 0.01 versus control group, ^**^ *p* < 0.01 versus model group by One‐way ANOVA with Tukey's test. (B) Nuclear translocation of NF‐κB p65 and expression of p‐STAT3 (Tyr705) were visualized using confocal microscopy (600×, LSM880, Carl Zeiss, Germany).

Given its consistent inhibition of TNF‐α and IL‐6 production in macrophages and its pronounced effects on neutrophil recruitment in vivo, BLH‐1 was selected for mechanistic investigation. As shown in Figure 3, BLH‐1 markedly reduced the phosphorylation of IκBα and STAT3 and inhibited the nuclear translocation of NF‐κB p65 in LPS‐stimulated RAW 264.7 cells. These results indicate that the anti‐inflammatory activity of BLH‐1 is associated with suppression of the NF‐κB and STAT3 signaling pathways. In in vivo assays assessing neutrophil activation, the tested compounds showed less potency than DEX, a clinically established anti‑inflammatory glucocorticoid. Nevertheless, they still markedly inhibited NF‑κB and STAT3 signaling activation. In future studies, additional inflammatory markers could be measured to identify the differences compared with DEX.

## 4 Conclusion

This study aimed to isolate the bioactive components of borojo and investigate their anti‐inflammatory activities. The results revealed the isolation of three new cyclopentenes (**BLH‐1∼BLH‐3**), a new fused ring compounds (**BLH‐4**), and five known compounds (**BLH‐5∼BLH‐9**). **BLH‐1**, **‐5**, and **BLH‐9** exhibited significant inhibitory effects on TNF‐α and IL‐6 release. Additionally, **BLH‐1, ‐2, ‐4, ‐6, ‐7** reduced the recruitment of neutrophils induced by LPS, CuSO_4_, and tail transection. Furthermore, the significant inhibitory effects of **BLH‐1** on the NF‐κB and STAT3 pathways suggest that bioactive components from borojo are effective in reducing inflammation. These findings imply that these compounds could serve as natural anti‑inflammatory agents for potential application in functional foods.

## Author Contributions

**Jinyin Ou**, **Zibin Lu**, and **Lifang Zou**: data curation, formal analysis, investigation, validation, writing – original draft. **Yuanru Zheng**: data curation, formal analysis. **Xuan Zhao**: data curation, methodology. **Xiduan Wei**: methodology. **Linzhong Yu**: conceptualization. **Huihui Cao**: methodology. **Huhu Zeng**, **Chunlin Fan**, and **Junshan Liu**: conceptualization, funding acquisition, supervision, writing – review and editing.

## Funding

This work was financially supported by the Guangdong Province Universities and Colleges Pearl River Scholar Funded Scheme (GDHVPS2018), the Young Elite Scientists Sponsorship Program by CACM (2019‐QNRC2‐C14), the Scientific Research Project of Guangdong Provincial Bureau of Traditional Chinese Medicine (20251243), and the National Key R&D Program of China (2017YFC1703800).

## Conflicts of Interest

The authors declare no conflicts of interest.

## Supporting information



This supplementary information includes NMR spectra (1H NMR, 13C NMR, DEPT, COSY, HMBC, and NOESY) of the compounds BLH‐1 to BLH‐4, the structures of the epimerides, and in vitro cytotoxicity test results on RAW 264.7 cells, as supporting data for the study.
**Supporting File 1**: cbdv71712‐sup‐0001‐SuppMat.docx.

## Data Availability

The data that support the findings of this study are available from the corresponding author upon reasonable request.
