# Supplementary material for: The Chemical Constituents From Borojo and Their Anti‐Inflammatory Activity
Source: Chem Biodivers. 2026 Sep 20;23(9):e71712. doi: 10.1002/cbdv.71712 (PMC13590131; doi:10.1002/cbdv.71712)
Supplement: Supplementary file 1 [file CBDV-23-e71712-s001.docx]

Fig. S1 ^1^H NMR spectrum of **BLH-1**

Fig. S2 ^13^C NMR and DEPT spectra of **BLH-1**

Fig. S3 Enlarged ^1^H-^1^H COSY spectrum of **BLH-1**

Fig. S4 Enlarged HMBC spectrum of **BLH-1**

Fig. S5 ^1^H NMR spectrum of **BLH-2**

Fig. S6 ^13^C NMR and DEPT spectra of **BLH-2**

Fig. S7 Enlarged ^1^H-^1^H COSY spectrum of **BLH-2**

Fig. S8 Enlarged HMBC spectrum of **BLH-2**

Fig. S9 Structure of the epimerides

Fig. S10 ^1^H NMR spectrum of **BLH-3**

Fig. S11 ^13^C NMR and DEPT spectra of **BLH-3**

Fig. S12 Enlarged ^1^H-^1^H COSY spectrum of **BLH-3**

Fig. S13 Enlarged HMBC spectrum of **BLH-3**

Fig. S14 ^1^H NMR spectrum of **BLH-4**

Fig. S15 ^13^C NMR and DEPT spectra of **BLH-4**

Fig. S16 Enlarged ^1^H-^1^H COSY spectrum of **BLH-4**

Fig. S17 Enlarged HMBC spectrum of **BLH-4**

Fig. S18 Enlarged NOESY spectrum of **BLH-4**

Fig. S19 Key ^1^H-^1^H COSY and HMBC correlations of **BLH-1**

Fig.S20 Key ^1^H-^1^H COSY and HMBC correlations of **BLH-2**

Fig. S21 Key ^1^H-^1^H COSY and HMBC correlations of **BLH-3**

Fig. S22 Key ^1^H-^1^H COSY and HMBC correlations of **BLH-4**

Fig. S23 the parent skeleton of cyclopentene

Fig. S24 the parent skeleton of fused-ring cyclopentenes

Table S1 structural comparison between BLH-1~3 and homologous cyclopentene

| No. | Compound | R_1_ | R_2_ | R_3_ | Source |
| --- | --- | --- | --- | --- | --- |
| 1 | BLH-1 | COOH | COOH | CH_2_COOCH_3_ | *B*. *patinoi* |
| 2 | BLH-2 | COOH | COOH | CH_3_ | *B*. *patinoi* |
| 3 | BLH-3 | COOH | COOH | CH_2_COOH | *B*. *patinoi* |
| 4 | 4-Cyano-cyclopent-1-enecarboxylic acid | COOH | H | CN | Chemical synthesis |
| 5 | cyclopent-3-ene-1,3-dicarboxylic acid 1-butyl ester | COOH | H | COOC_4_H_9_ | Chemical synthesis |
| 6 | Cyclopent-3-ene-1,3-dicarboxylic acid 3-ethyl ester | COOC_2_H_5_ | H | COOH | Chemical synthesis |
| 7 | 1-Cyclopentene-1,4-dicarboxylic acid | COOH | H | COOH | Chemical synthesis |
| 8 | 3-O-ethyl 1-O-(2-methylpropyl) (1R)-cyclopent-3-ene-1,3-dicarboxylate | COOC_2_H_5_ | H | COOCH_2_CH(CH_3_)_2_ | Chemical synthesis |
| 9 | Diethyl cyclopent-3-ene-1,3-dicarboxylate | COOC_2_H_5_ | H | COOC_2_H_5_ | Chemical synthesis |
| 10 | Diethyl (1R)-cyclopent-3-ene-1,3-dicarboxylate | COOC_2_H_5_ | H | COOC_2_H_5_ | Chemical synthesis |
| 11 | (1R,2R)-4-methylcyclopent-3-ene-1,2-dicarboxylic acid | CH_3_ | COOH | COOH | Chemical synthesis |
| 12 | 4-Methylcyclopent-3-ene-1,2-dicarboxylic acid | CH_3_ | COOH | COOH | Chemical synthesis |
| 13 | Dimethyl 4-methylcyclopent-3-ene-1,2-dicarboxylate | CH_3_ | COOCH_3_ | COOCH_3_ | Chemical synthesis |
| 14 | Diethyl 4-methyl-3-cyclopentene-1,2-dicarboxylate | CH_3_ | COOC_2_H_5_ | COOC_2_H_5_ | Chemical synthesis |
| 15 | 3-Cyclopentene-1,2-dicarboxylic acid, 4-methyl-, dimethyl ester, trans- | CH_3_ | COOCH_3_ | COOCH_3_ | Chemical synthesis |
| 16 | Bis(2-methylpropyl) 4-methylcyclopent-3-ene-1,2-dicarboxylate | CH_3_ | COOCH_2_CH(CH_3_)_2_ | COOCH_2_CH(CH_3_)_2_ | Chemical synthesis |
| 17 | Dimethyl cyclopent-3-ene-1,2-dicarboxylate | H | COOCH_3_ | COOCH_3_ | Chemical synthesis |
| 18 | Diheptyl cyclopent-3-ene-1,2-dicarboxylate | H | COOC_7_H_15_ | COOC_7_H_15_ | Chemical synthesis ^[6]^ |
| 19 | Diheptyl 4-methylcyclopent-3-ene-1,2-dicarboxylate | CH_3_ | COOC_7_H_15_ | COOC_7_H_15_ | Chemical synthesis |
| 20 | Dihexyl cyclopent-3-ene-1,2-dicarboxylate | H | COOC_6_H_13_ | COOC_6_H_13_ | Chemical synthesis |

Table S2 Structural comparison between BLH-4 and homologous fused-ring cyclopentenes

| No. | Compound | R_1_ | R_2_ | Source |
| --- | --- | --- | --- | --- |
| 1 | BLH-4 | COOH | O | *B*. *patinoi* |
| 2 | (3aR,6aS)-6-(hydroxymethyl)-3,3a,4,6a-tetrahydrocyclopenta[b]furan-2-one | CH_2_OH | O | Chemical synthesis |

Table S3. The cytotoxicities on RAW 264.7 cells (*n*=3)

| No. | Concentration | Survival (%) |
| --- | --- | --- |
| **Control** | / | 100.27 ± 3.51 |
| **BLH-1** | 20 μM | 98.773±1.367 |
|  | 10 μM | 103.264±1.574 |
| **BLH-2** | 20 μM | 94.685±2.122 |
|  | 10 μM | 94.251±1.664 |
| **BLH-3** | 20 μM | 101.814±1.003 |
|  | 10 μM | 113.231±3.677^*^ |
| **BLH-4** | 20 μM | 105.682±10.173 |
|  | 10 μM | 100.639±8.202 |
| **BLH-5** | 20 μM | 100.247±1.287 |
|  | 10 μM | 102.850±1.645 |
| **BLH-6** | 20 μM | 98.576±9.311 |
|  | 10 μM | 92.709±12.205 |
| **BLH-7** | 20 μM | 85.871±5.362^*^ |
|  | 10 μM | 93.795±11.777 |
| **BLH-8** | 20 μM | 112.338±1.375^*^ |
|  | 10 μM | 102.651±1.295 |
| **BLH-9** | 20 μM | 94.913±6.278 |
|  | 10 μM | 111.799±1.311^*^ |
